# Prevalence of gestational diabetes and associated risk factors in the population of Safi Province in Morocco

**DOI:** 10.11604/pamj.2020.37.281.21798

**Published:** 2020-11-26

**Authors:** Hamid Chamlal, Mohamed Mziwira, Mohammed El Ayachi, Rekia Belahsen

**Affiliations:** 1 Laboratory of Biotechnology, Biochemistry and Nutrition, Training and Research Unit on Nutrition and Food Sciences, Faculty of Sciences, Chouaib Doukkali University, El Jadida, Morocco,; 2 Higher Normal School of Hassan II University, Casablanca, Morocco

**Keywords:** Gestational diabetes, prevalence, risk factors, Safi, Morocco

## Abstract

**Introduction:**

the association of diabetes with pregnancy is a frequent gestational situation that constitutes a real public health problem with prevalence and risk factors varying according to the population type. The present study aims to determine the prevalence of gestational diabetes (GD) and associated risk factors (socio-demographic characteristics, medical and obstetric history) in pregnant women from the Moroccan province of Safi.

**Methods:**

this cross-sectional, descriptive and analytical study was conducted during the period from October 2018 to February 2019, on 208 pregnant women, attending the antenatal consultation services of four health centers equally distributed between urban and rural areas of Safi Province in Morocco. The data on blood glucose levels and the risk factors for gestational diabetes generally described in the literature were collected. The statistical Chi-square test and the logistic regression were used to check the association between gestational diabetes and the risk factors.

**Results:**

the mean age of the pregnant women sample was 28.9 ± 6.8 years and the prevalence of GD was 24.5%. Using multivariate logistic regression, the most significantly associated risk factors were the diabetic inheritance of a primary relative (OR=2.616 [95% CI: 1.281-5.341]; p=0.008) and the high education level (OR=2.301 [95% CI: 1.123-4.716] p=0.023).

**Conclusion:**

the results show that gestational diabetes is a frequent pathology among the study population. The data revealed also that the diabetes inheritance (primary relative) and the high level of education are the main risk factors of this pathology in the surveyed pregnant women in the study province.

## Introduction

Diabetes is a major public health problem around the world [1], characterized by a number of cases in continuous increase in the last decades [2]. It is estimated as 463 million adults (1 in 11 people) and the figure is expected to reach 578 million by 2030 [3]. Indeed, diabetes is becoming more and more a chronic disease affecting people not only in high-income countries but also progressively in low and middle-income countries, where raising awareness on the diagnosis and the risk factors as well as the access to care are still limited [4]. The emergence of this pathology has been related to changes in lifestyle [5], urbanization [6], lack of physical activity [7], obesity and high blood pressure. Diabetes is one of problems among the epidemiological transition with enormous health and economic consequences, posing new challenges to the national health systems [8]. Gestational diabetes is a of type of diabetes characterized by glucose tolerance disorder leading to variable hyperglycemia, starting or diagnosed for the first-time during pregnancy regardless of the treatment required and the evolution of the post-partum [9,10].

GD affects more than 135,000 women each year, representing 1% to 14% of all pregnancies [11]. It is a public health problem associated with many complications during pregnancy or at birth in both, the mother and the child and in the short and long term [12]. The main factors linked to GD are manifold. The maternal age from 35 years, obesity, ethnicity, diabetes family history of primary relative, personal history of GD, fetal death in utero or macrosomia and the polycystic ovary syndrome have been reported in the literature [5,13]. The multiplicity of the risk factors and their interactions makes difficult the prediction of individual risk [5]. The prevalence of GD is difficult to estimate. It varies widely around the world [14] and sometimes even within the same country, depending on the ethnic origin [5,15]. It is still difficult to obtain in certain African countries because of material difficulties [16]. GD prevalence depends on the screening method (universal or targeted), the diagnosis criteria used and the prevalence of the type 2 diabetes in each country [5].

In Morocco, gestational diabetes affects 1 in 10 pregnant women. However, except in the university environment, knowledge about its screening and the practices of its management are limited [4]. In some mono-centric and regional studies, the reported prevalence varies between 7.7% [17] and 24.2% [4]. Given the prevalence of type2 diabetes and obesity, this prevalence may be even higher in Morocco [17]. The comprehensive care of pregnant women in Morocco is provided during antenatal consultations carried out in primary care centers, which represent the main sites of seeking prenatal care and the prevention and surveillance of GD [3]. Nevertheless, the awareness messages of the different protocols are not uniform with very limited information to apply them in practice [4], which can affect the health of the mother-child couple. In view of the above, this study aims to determine the prevalence of GD and associated risk factors, in pregnant women in the Moroccan province of Safi.

## Methods

The study is a cross-sectional, descriptive and analytical survey carried out in four health centers selected according to the rate of antenatal consultation (ANC) and the geographical situation distributed equally between urban and rural areas. These are public health units offering a diverse set of preventive care and representing the first resort for pregnant women to access prenatal consultations in the public sector. The study was carried out over a period of five-month from October 2018 to February 2019 and targeted pregnant women attending ANC services in the four selected health centers (HCs). The study population included all pregnant women over the age of 17.

Sampling protocol was carried out in two phases, in the first stage, we choose the health centers frequented by pregnant women in the study province, these HCs are selected according to the rate of antenatal consultation (ANC) exceeding 80% of the expected births per semester, their geographical location covering the representative majority of the province and their distribution between urban and rural areas. In a 2^nd^ stage, the sampling consisted of randomly selecting all pregnant women attending designated health centers. The number of these pregnant women achieved 389. We excluded pregnant women with one of the following: history of gestational diabetes, pre-existing diabetes, another chronic disease, high-risk pregnancy, no follow up file. The final sample size was 208 pregnant women.

Data were collected using a questionnaire, adapted and supplemented from our previous studies and the reliable reference literature on gestational diabetes. It was then pre-tested and validated by an expert committee. The participants were informed beforehand about the survey objectives and gave their informal consent before their inclusion in the study sample and the data were treated in a fair and equitable manner with respect of confidentiality [18]. For the screening of diabetes, the capillary fasting glucose (FG) test [19] and the postprandial glucose test (PPG) two hours after a standard meal were used [20]. The measurements were made by last generation readers. The diagnosis was made on the basis of FG ≥0.92g/L value for pregnant women less than 12 weeks of gestation. For those who exceed the first trimester, FG ≥0.92g/L and/or a PPG ≥1.20g/L were used. Abnormal values of glycemia were used as proposed by the International Association of Diabetes and Pregnancy Groups (IADPSG) and the World Health Organization (WHO) criteria [21].

Information on socio-demographic characteristics and socio-economic level including age, area of residence, education level, paid jobs and income are also collected using questionnaires. In addition, information on the history of blood pressure problem, primary relative diabetic relatives (father or mother or both) and the body mass index (BMI) are recorded. Data concerning the obstetric history, the parity, the history of miscarriage, cesarean surgery, birth defects, polycystic ovary syndrome (PCOS), macrosomia and the complications during childbirth were also collected. Statistical analyses were undertaken using Soft Package Social Sciences (SPSS) software version 23.0. The Chi^2^ test was used to compare the results. The risk was assessed using logistic regression analysis and adopting odds ratio (OR) estimates and confidence interval (95% CI) with p values <0.05, considered as statistically significant.

## Results

Table 1 presents the data concerning the socio-demographic characteristics of the surveyed pregnant women. The table shows that the women age was ranging from 17 to 43 years, with an average of 28.9±6.8 years and with women over 35 years representing 21.2% and 61.1% among them from rural area. Almost all of the study pregnant women (96.2%) were housewives, with no paid employment and 50% of them from low socio-economic level. The prevalence of gestational diabetes mellitus in the study sample was 24.5%. In addition, the results show that high education level was associated with the GD (p=0.004). In Table 2, the data on the medical history show that diabetic inheritance of a primary relative (p=0.001) and the history of blood pressure problems (p=0.030) are the medical parameters significantly associated with the onset of gestational diabetes in this population.

**Table 1:** socio-demographic characteristics associated with gestational diabetes in pregnant women attending ANC services in the province of Safi

Socio-demographic characteristics	Total n=208	Without GD n=157	With GD n=51	P value
	n (%)	n (%)	n (%)	
**Age: mean 28.9 years, standard deviation 6.8 years**				
≥35 years	44(21,2)	32(20,4)	12(23,5)	0,633
(17- 35) years	164(78,8)	125(79,6)	39(76,5)	
**Origin**				
Rural	127(61,1)	100(63,7)	27(52,9)	0,171
Urban	81(38,9)	57(36,3)	24(47,1)	
**Educational level**				
<7ans	158(76,0)	127(80,9)	31(60,8)	0,004
≥7ans	50(24,0)	30(19,1)	20(39,2)	
**Paid occupation**				
Yes	8(3,8)	5(3,2)	3(5,9)	0,384
No	200(96,2)	152 (96,8)	48(94,1)	
**Socio-economic level (income in MDH/month)**				
Low (<3000)	105(50,5)	73(46,5)	32(62,7)	0,130
Middle (3000-5000)	97(46,6)	79(50,3)	18(35,3)	
High (>5000)	6(2,9)	5(3,2)	1(2,0)	

GD: gestational diabetes ; MDH: Moroccan Dirham

**Table 2:** medical history factors associated with gestational diabetes in pregnant women attending ANC services in the province of Safi

Medical history	Total n=208	Without GD n=157	With GD n=51	P value
	n (%)	n (%)	n (%)	
**Blood pressure problem**				
Yes	33(15,9)	20(12,7)	13(25,5)	0.030
No	175 (84,1)	137(87,3)	38(74,5)	
**Family history of diabetes (primary relative)**				
Yes	52(25)	30(19,1)	22(43,1)	0,001
No	156(75)	127(80,9)	29(56,9)	
**Body mass index**				
≥30	90 (43,3)	67 (42,7)	23(45,1)	0,762
<30	118 (56,7)	90 (57,3)	28(54,9)	

GD: gestational diabetes

The Table 3 gathers the results on the study women obstetric history. No significant association was found between the studied factors and the appearance of GD. The Table 4 presents the results of the univariate analysis of the factors associated with gestational diabetes, using the simple logistic regression. The results show a significant association of the higher education level (OR=2.731 [95% CI: 1.372-5.438] p=0.004), the history of blood pressure problem (OR=2.343 [95% CI: 1.069-5.139] p=0.034) and the heredity (primary diabetic relatives) (OR=3.211 [95% CI: 1.623-6.353] p=0.001) with gestational diabetes.

**Table 3:** obstetric history factors associated with GD in pregnant women attending ANC services in the province of Safi

Obstetric history	Total n=208	Without GD n=157	With GD n=51	P value
	n (%)	n (%)	n (%)	
**Parity**				
Multiparous	129(62)	96(61,1)	33(64,7)	0,649
Primiparous	79(38)	61(38,9)	18 (35,3)	
**History of miscarriage**				
Yes	54(26)	42(26,8)	12(23,5)	0,648
No	154(74)	115(73,2)	39(76,5)	
**History of caesarean section**				
Yes	15(7,2)	9(5,7)	6(11,8)	0,148
No	193(92,8)	148(94,3)	45(88,2)	
**History of birth defects**				
Yes	4(1,9)	4(2,5)	0(0,0)	0,250
No	204(98,1)	153(97,5)	51(100,0)	
**History of polycystic ovary syndrome**				
Yes	16(7,7)	10(6,4)	6(11,8)	0,209
No	192(92,3)	147(93,6)	45 (88,2)	
**History of macrosomia**				
Yes	10(4,8)	5(3,2)	5(9,8)	0,055
No	198(95,2)	152(96,8)	46(90,2)	
**History of complications during childbirth**				
Yes	13(6,3)	8(5,1)	5(9,8)	0,227
No	195(93,8)	149(94,9)	46(90,2)	

GD: gestational diabetes

**Table 4:** simple logistic regression analysis of the factors associated with GD

Independent variables	Odds ratio	Confidence interval 95%		P value
		Lower	Higher	
**Education level**				
<7ans	1			
≥7ans	2,731	1,372	5,438	0,004
**History of blood pressure problem**				
No	1			
Yes	2,343	1,069	5,139	0,034
**Diabetic inheritance of a 1^st^ degree**				
No	1			
Yes	3,211	1,623	6,353	0,001

The association between the factors studied and gestational diabetes using multivariate analysis is presented in Table 5. The analysis showed a significant association of higher education level (OR=2.301 [95% CI: 1.123-4.716] p=0.023) and the heredity (primary diabetic relative) (OR=2.616 [95% CI: 1.281-5.341] p=0.008) with gestational diabetes.

**Table 5:** multiple logistic regression analysis of the factors associated with GD

Independent variables	Odds ratio	Confidence interval 95%		P value
		**Lower**	**Higher**	
**Education level**				
<7ans	1			
≥7ans	2,301	1,123	4,716	0,023
**History of blood pressure problem**				
No	1			
Yes	1,760	0,760	4,076	0,187
**Diabetic inheritance of a 1^st^ degree**				
No	1			
Yes	2,616	1,281	5,341	0,008

## Discussion

The present study reports a prevalence of 24.5% of gestational diabetes in pregnant women attending ANC in Safi, a coastal province of Morocco. The disease was found in the women aged 35 years or more, mostly illiterate, from rural areas, low socio-economic status and without paid employment. Moreover, at present, there is no international consensus on the screening strategies, as well as on the diagnostic tools or even on the thresholds to use for gestational diabetes [21-23]. This has led to a controversial situation resulting in a great heterogeneity of international recommendations and practices [23,24]. In the present study, the screening of GD was based on fasting blood glucose level or postprandial blood glucose level two hours after a standard meal, generally carried out in the morning after breakfast. The use of this so-called alternative approach [23] takes into account certain characteristics that are potential confounding factors in Morocco such as the area of residence, education level, affordability and geographic access to blood tests [4]. The use of blood glucose meters for example allows easier test and immediate results at low cost [23]. In addition, Anglo-Saxon guidelines recommend systematic screening for gestational diabetes by performing fasting or postprandial blood glucose levels during the first antenatal consultation and at the 28^th^ week of amenorrhea respectively [23,25]. Furthermore, the latest recommendations from the international federation of gynecology and obstetricians advised adopting accessible approaches, especially in low- and middle-income countries [26].

To this end, capillary test screening is recommended at primary health facilities level, which could help reducing delays and an early detection of GD limiting negative results during pregnancy, especially in environments where access to laboratories is an obstacle [4]. In our context, women who have higher blood glucose levels, are referred to physicians in health centers for a confirmatory oral hyperglycemia test in the aim to avoid false diabetics. Screening for diabetes during pregnancy certainly has both maternal and fetal benefits with the short-term goal of preventing fetal macrosomia and associated obstetric accidents [14]. Finally, setting up a monitoring system for mothers and newborns would help preventing obesity and diabetes [12]. The usefulness of screening varies depending on the initial characteristics of the population and the economic impact of health in each country [21].

The reported prevalence of 24.5% of GD in the present study on pregnant women is comparable to that found in the few single-center studies available in different Moroccan regions. In fact, this prevalence is the same as that recorded in the region of Marrakech and Al Haouz (24.2%) [4], but much higher than that found at the military hospital of Rabat (7.7%) [17] and (8.2%) [27]. Compared to international data, our result is close to the prevalence found in South Africa (25.8%) [16], but higher than those found in Guinea (16.3%) [25], in Algeria (14.6%) [22] and in the Democratic Republic of Congo (11.3%) [14]. The prevalence of GD in the present study, however, is lower than that of a similar study conducted in Senegal (33.1%) [28]. This divergence of the results reported in all these studies could be explained by the different characteristics of the populations studied and by the different screening criteria used.

The main factors associated with GD reported in the literature [19,21,23] are of socio-demographic, medical and obstetric history types. Among these factors, maternal age of 35 years and over, the history of blood pressure problems, the family history of primary relative diabetes (father or mother or both), obesity (BMI ≥30kg/m^2^), the history of fetal malformations or macrosomia (≥4kg), caesareans and the complications during delivery are reported. Other risk factors with moderate influence such as ethnicity [5,15] and the polycystic ovary syndrome [5] are also found to be associated with GD. In the present study, pregnant women with gestational diabetes were over 35 years of age, the majority was from rural areas and almost all were of low socio-economic status. They were housewives with no income and mostly illiterate. The level of education indeed corresponded to the characteristics significantly associated with diabetes in this population. However, contrary to what is expected, it is the high level of education that is associated with GD in this population. The social vulnerability of the Moroccan female population has already been demonstrated at the national level, by the high commission for planning of Morocco, estimating the multidimensional poverty rate at 14.7% in 2014 with a rate of 41.9% of illiteracy, especially in rural areas [29].

The medical history and heredity of diabetes (primary relative history of diabetes) were also significantly associated with gestational diabetes in the pregnant study population. This result is corroborated by several studies on the African continent such as in the Democratic Republic of Congo [16] or in the same North African region in Algeria [30] as well as in the same country [17,27]. Theoretically, the prevalence of GD in a population is even higher than that of type 2 diabetes [23]. In Morocco, the prevalence of the latter is high, going from 6.6% in 2005 [31] to 10.6% in 2018 [32]. Moreover, this prevalence of diabetes, being estimated at 7% in the region of the population studied which is the “Marrakech-Safi” region [33], the parameter of family history of diabetes, could be one of the risk factors for the development of GD [34]. This observation has led some authors to consider Maghreb populations at high risk of GD requiring systematic screening [20].

Another medical factor examined here, is the problem of the history of blood pressure, also significantly associated with GD in this population of pregnant women. Blood pressure problems are usually linked to pregnancy-induced hypertension or pre-eclampsia, one of the causes of high-risk pregnancies that endanger the life of the mother-newborn couple and which is very common in women with gestational diabetes [23]. Moreover, given the high prevalence of blood pressure, estimated as 9.7% in the “Marrakech-Safi” study region, could be very detrimental to this population [33].

Another factor reported as one of the main risk factors for GD is obesity [24,35]. However, no association of this problem with this disease was found in the present investigation. This result is probably linked to the already widespread obesity, which affects a high proportion of Moroccan women over the age of 18 [36]. It may also be related to characteristics of the study population that differ from those reported in the literature and to the size of the study sample. Furthermore, the data from this study did not show any significant association of the obstetric variables examined with GD. This result disagrees with a previous study on 100 cases of Moroccan women [37], reporting that obstetric history was the most predominant risk factor for GD in addition to heredity of diabetes and obesity.

## Conclusion

The data from this study show an alarming prevalence of gestational diabetes affecting about 1/4 of the surveyed population. Heredity of diabetes (primary parent's history of diabetes) and high level of education were the main risk factors for gestational diabetes in the study population. Despite the limitations of the present study, these results made it possible to have a critical profile of this vulnerable population. Moreover, as at the international level, the study data confirm that gestational diabetes is also a local health problem, with an alarming increase accentuated by under-diagnosis, prevalent in Morocco as in other countries with low- and middle-income. The study also underlines the need to focus the efforts of decision-makers on the management of GD to prevent maternal-fetal complications. This can be achieved by raising awareness among key actors, especially at the primary care level, on the importance of early detection of diabetes and by sensitizing pregnant women to risk factors associated with GD to ensure adequate management.

### What is known about this topic

- Gestational diabetes is a public health problem anywhere in the world;
- Lack of international consensus on methods and testing for gestational diabetes;
- Rarity of Moroccan studies on the prevalence of gestational diabetes and associated risk factors and lack of data among pregnant women in the study province (Safi).

### What this study adds

- Gestational diabetes represents an alarming situation affecting about 1/4 of the Moroccan pregnant women;
- The primary relative family link of the diabetes and high education are risk factors for gestational diabetes in the studied pregnant women;
- Poor management of GD, especially in terms of diagnosis, can affect maternal and fetal health at the level of health centers in the studied province.
